# The Medical News Visiting List for 1903

**Published:** 1902-12

**Authors:** 


THE MEDICAL NEWS VISITING LIST FOR 1903. Weekly (dated, for 30 patients); monthly (undated, for 120 patients per month); perpetual (undated, for 30 patients weekly per year); and perpetual (undated, for 60 patients weekly per year). The first three styles contain 32 pages of data and 160 pages of blanks.

The 60-patient perpetual consists of 256 pages of blanks. Each style in one wallet-shaped book, with pocket, pencil and rubber. Seal grain leather, $1.25. Thumb-letter index, 25 cents extra. Lea Brothers & Co., Publishers, Philadelphia and New York.

The publisher says: "A visiting list is an indispensable convenience for the active practitioner. Its carefully adapted blanks enable him at once to note clinical details of every day work, as well as charges and receipts, and to unburden his memory of that which can be better carried on paper." The "Medical News Visiting List" presents some points of advantage over most publications of the sort, not the least of which is that, being printed on thin high grade paper, it takes up very little room in the pocket. In addition to the blank pages arranged for keeping a record of visits, engagements, addresses of patients and nurses, and a cash account, it contains thirty-two pages of valuable data covering remedies, table of doses, poisons and antidotes, examination of urine, etc. R.

